# Chemical Properties of Human Dentin Blocks and Vertical Augmentation by Ultrasonically Demineralized Dentin Matrix Blocks on Scratched Skull without Periosteum of Adult-Aged Rats

**DOI:** 10.3390/ma15010105

**Published:** 2021-12-24

**Authors:** Bowen Zhu, Kenji Yokozeki, Md. Arafat Kabir, Masahiro Todoh, Toshiyuki Akazawa, Masaru Murata

**Affiliations:** 1 Division of Oral Regenerative Medicine, School of Dentistry, Health Science University of Hokkaido, Kanazawa 061-0293, Japan; zhubear@hoku-iryo-u.ac.jp (B.Z.); yokozeki@hoku-iryo-u.ac.jp (K.Y.); kabiranan@yahoo.com (M.A.K.); 2 Biomechanical Design Faculty of Engineering, Hokkaido University, Kita 13, Nishi 8, Kita-ku, Sapporo 061-0819, Japan; todoh@eng.hokudai.ac.jp; 3 Industrial Technology and Environment Research Development, Hokkaido Research Organization, Kita 19-jo Nishi 11-chome, Kita-ku, Sapporo 060-0819, Japan; akazawa-toshiyuki@hro.or.jp

**Keywords:** dentin-derived biomaterials, vertical augmentation, demineralized dentin matrix, bone regeneration, human dentin, ultrasonic scratch

## Abstract

Vertical augmentation is one of the most challenging techniques in bone engineering. Several parameters, such mechano-chemical characteristics, are important to optimize vertical bone regeneration using biomaterials. The aims of this study were to chemically characterize human dentin blocks (calcified demineralized dentin matrix: CDM, partially demineralized dentin matrix: PDDM and completely demineralized dentin matrix: CDDM) (2 × 2 × 1 mm^3^) chemically and evaluate the behavior of PDDM blocks on non-scratched or scratched skulls without periosteum of adult rats (10–12 months old, female) as a vertical augmentation model. The dissolved efficiency of CDM showed 32.3% after ultrasonic demineralization in 1.0 L of 2% HNO_3_ for 30 min. The 30 min-demineralized dentin was named PDDM. The SEM images of PDDM showed the opening of dentinal tubes, nano-microcracks and the smooth surface. In the collagenase digestion test, the weight-decreasing rates of CDM, PDDM and CDDM were 9.2%, 25.5% and 78.3% at 12 weeks, respectively. CDM inhibited the collagenase digestion, compared with PDDM and CDDM. In the PDDM onlay graft on an ultrasonically scratched skull, the bone marrow-space opening from original bone was found in the bony bridge formation between the human PDDM block and dense skull of adult senior rats at 4 and 8 weeks. On the other hand, in the cases of the marrow-space closing in both non-scratched skulls and scratched skulls, the bony bridge was not formed. The results indicated that the ultrasonic scratching into the compact parietal bone might contribute greatly to the marrow-space opening from skull and the supply of marrow cells, and then bony bridge formation could occur in the vertical augmentation model without a periosteum.

## 1. Introduction

The recent popularity of implant dentistry has led to an increasing demand for alveolar bone regeneration. Autogenous bone grafts are still considered as gold standard for bone augmentation because of the excellent osteoinductivity and osteoconductivity [1,2], but it has some impediments such as limited availability and donor site morbidity. Therefore, the search for osteoinductive biomaterials always remains in the spotlight [3].

Extracted human teeth are generally discarded as infectious medical waste throughout the world. However, the dental community is now considering using human permanent and milk teeth as a native resource for patients and their family. Highly calcified tissues such as cortical bone and dentin do not perform better in both osteoinduction and bone formation than spongy bone, decalcified bone matrix (DBM) and demineralized dentin matrix (DDM) [4]. Dentin and bone are mineralized tissues and almost identical in their chemical components. Dentin and bone consist of 18% collagen, 70% apatite, 10% body fluid and 2% non-collagenous proteins (NCPs), including a small amount of growth factors such as bone morphogenetic proteins (BMPs), transforming growth factor-β, insulin-like growth factor and basic fibroblast growth factor [5,6].

In 1967, the bone-inducing property of rabbit and rat dentin was discovered in the intramuscular pockets [7,8]. Additionally, the osteoinductive property of human DDM granules was found at 4 weeks after implantation in the subcutaneous tissue of 4-week-old male athymic mice [3]. Human DDM and DBM can be defined as acid-insoluble collagen binding several growth factors including BMPs [9,10]. BMPs are known to exist in the bone/dentin matrix and osteosarcoma tissue, functioning to differentiate perivascular mesenchymal stem cells into cartilage and bone tissues [11,12]. Type I collagen occupies about 90% of the organic parts of tissues, with the rest comprised of non-collagenous proteins (NCPs), biopolymers, lipid, citrate, lactate, etc. NCPs include phosphophoryn, sialoprotein, glycoprotein, proteoglycan, osteopontin (OPN), osteocalcin and dentin matrix protein 1. These proteins are known to trigger the bone resorption and generation processes [13,14,15,16]. 

Human vital teeth are a rich source of stem cells, matrices, trace metal ions and growth factors. Despite this fact, extracted non-functional teeth are routinely discarded as potentially infectious medical waste. However, we have noted the bone-inducing property of dentin and have been studying the medical recycling of human teeth as novel graft material for bone regeneration [9,17]. In 2010, human vital wisdom tooth-derived DDM granules induced bone and cartilage independently at 4 weeks in the subcutaneous tissues of nude mice, and the sequence for bone induction was similar to that of DBM granules [18]. In 2017, a root-form of partially demineralized human dentin matrix (PDDM) was grafted into iliac bone critical defects in adult sheep, and X-ray CT images revealed the absorption of PDDM and bone ingrowth into pores and the original pulp space at 2 and 4 months. The root-form of PDDM had a unique structure with micro-nano cracks, dentinal tube spaces and artificial pores [19]. The acid solution for the PDDM root block was 2% HNO_3_, and the ultrasonic demineralization time was 30 min from a viewpoint of clinical application. The surface area and the porous structure of the 3D interconnection in scaffolds are known to be major factors in achieving better cellular performance in bone induction and conduction. Once dentin is demineralized, the dentinal tubes become wider and expose dense collagen fibers as a scaffold and network, serving as a channel for the release of these essential proteins; these proteins might show either osteoconductive or osteoinductive properties [5,20,21]. In 2021, rat dense parietal bone scratched by an ultrasonic scalar tip using acidic electrized water (pH 2.6) induced new bone at 3 weeks in subcutaneous tissues, while the rat fresh parietal bone without scratching failed to induce bone, and cartilage bone induction occurs mainly in micro-damaged areas [22]. An in vitro study using electron microscopy revealed the superior attachment of osteoblast-like cells to the surface of DDM, compared to that of mineralized dentin [23].

Vertical augmentation is one of the most challenging techniques in bone engineering. Several parameters, such mechano-chemical characteristics, are important to optimize vertical bone augmentation using biomaterial scaffolds. Although there have been many studies employing chemical tests on bone, human dentin blocks have been little investigated. Moreover, almost all animal studies are involved in bone defect (inlay) models. The chemical properties of several kinds of dentin blocks have not been studied well, and vertical augmentation (onlay) models without a periosteum using PDDM blocks has not been performed until now. In the present onlay model, we focused on PDDM block demineralized ultrasonically in 1.0 L of 2% HNO_3_ for 30 min, from a clinical viewpoint of the immediate DDM autograft after tooth extraction, as described previously [19].

The aims of this study were to chemically characterize human dentin block and evaluate the behavior of human PDDM block on 10–12-month-old rat skulls without a periosteum as a vertical augmentation model.

## 2. Materials and Methods

### 2.1. Preparation of Scaffolds 

**Figure 1:**
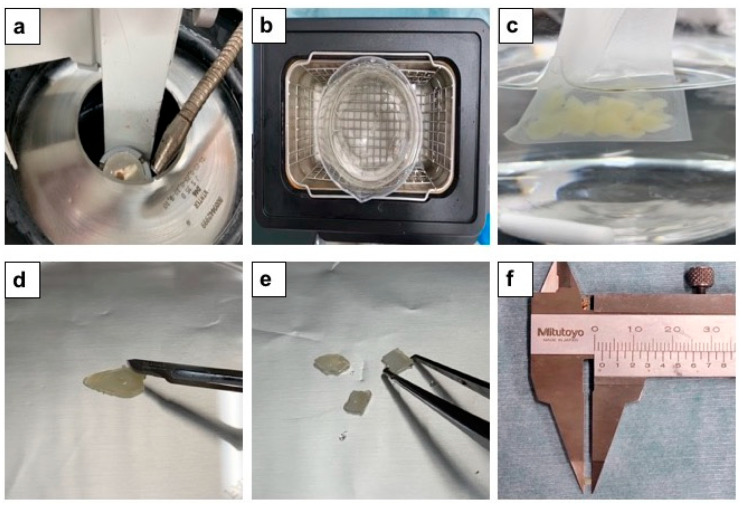
Preparation of human dentin blocks. (**a**) Dentin-root portion was sliced into several disks (1.0 mm in thickness) by using electrically rotating cutting machine (Leica SP1600 Germany), (**b**) Ultrasonic demineralization in 2% HNO_3_ liquid (1.0 L) for 30 min, (**c**) Ultrasonic washing in D.W. (1.0 L) for 30 min, (**d**–**f**) Demineralized dentin disks were cut into blocks (2 × 2 × 1 mm^3^).

This study received approval from Ethics Committee (No.165), Health Sciences University of Hokkaido, Hokkaido, Japan. The participants were made aware of the objectives and procedures of the study and agreed to participate by signing terms of informed consent. The scaffolds were prepared from human vital non-functional wisdom teeth. After soft tissues dissection and vigorous washing with distilled water (D.W.) (Otsuka Distilled water co., Tokyo, Japan), the extracted teeth were fixed in tooth-fixing device (Mr. Fix. Tokyo Iken Co. Ltd., Tokyo, Japan), crown and root portions were divided by a diamond disk bur (SHOFU Diamond disk 61 T, Ratingen, Germany) and the root portion was sliced into several dentin disks (1.0 mm in thickness) by using an electrically rotating cutting machine (Leica SP1600, Nussloch, Germany), and the disks were cut by scissors into 2 mm × 2 mm × 1 mm blocks for a calcified dentin matrix (CDM) group (Figure 1). For a partially demineralized dentin matrix (PDDM) group, the disks were ultrasonically demineralized at 120 W and 38 kHz for 30 min in 1.0 L of 2% HNO_3_ solution. All blocks were washed in 1.0 L of D.W. in ultrasonic machine for 30 min. For the completely demineralized dentin matrix (CDDM) group, the disks were ultrasonically demineralized for 3 days in 1.0 L of 2% HNO_3_ for 3 days at 25 °C under the automatically control (Powersonic 603, Hwashin Technology Company, Busan, Korea).

### 2.2. Microstructure Observation

The morphological characterization of the dentin blocks using a field emission scanning electron microscope (FE-SEM: JSM-7400F, JEOL, Tokyo, Japan) or a scanning electron microscope (SEM: S-3500N, HITACHI, Tokyo, Japan) were used to observe the surface texture and native geometrical tunnel structure of the dentin. The samples were fixed in 5% glutaraldehyde and 1% formaldehyde for 2 days. The dentin blocks were dehydrated in a graded ethanol series (20%, 40%, 60%, 80%, and absolute ethanol) for 10–15 min and placed in vacuum desiccators for overnight drying. The following day, samples were sputter coated with 10 nm of Au or Pt.

### 2.3. Dissolved Efficiency of Calcified Dentin Matrix (CDM) 

Four CDM blocks (size: 2 × 2 × 1 mm^3^) of each group (CDM, PDDM, CDDM) were selected for the dissolved efficiency test. CDM were dried in desiccator including silica gel for 30 min and weighed (A&D Electronic Balance ER-182A) before demineralization. CDM were treated with 1.0 L of 2% HNO_3_ by the ultrasonic machine (Powersonic 603, HSt Hwashin Technology, Company, Seoul, Korea) for 15 min (each number = 4). Each sample was washed in 1.0 L of D.W. by the ultrasonic machine for 15 min, dried in desiccator for 30 min and weighed. Next the samples were demineralized in 1.0 L of 2% HNO_3_ by the ultrasonic machine for 15 min, and each sample was washed in 1.0 L of D.W. by the ultrasonic machine for 15 min, dried in desiccator for 30 min and weighed. At last, the samples were demineralized in 1.0 L of 2% HNO_3_ by the ultrasonic machine for 15 min, each sample was washed in 1.0 L of D.W. by the ultrasonic machine for 15 min, dried in desiccator for 30 min and weighed. The procedure was carried out at room temperature. Gross-view, SEM and statistical analysis were performed.

### 2.4. Dentin Absorption Analysis by Collagenase Digestion

Four block samples (size: 2 × 2 × 1 mm^3^) of each group (CDM, PDDM, CDDM) were selected for the collagenase digestion test. Samples were freeze-dried before a treatment with bacterial collagenase from C. histolyticum (Funakoshi). Samples of dentin block were treated with 40 mL of collagenase solution (0.375 mg/mL D.W) in a rotary shaker (Rotary Shaker NA-301, Tokyo, Japan) at a speed of 4 rpm for 3, 6, 12 weeks (each number = 4). Next, each sample was washed in D.W. and freeze-dried. The procedure was carried out at room temperature. Gross-view, SEM and weights of freeze-dried CDM, PDDM and CDDM before and after the collagenase digestion were measured and statistical analysis was performed. 

### 2.5. Animal Experiment 

The animal experiment was examined by the Institutional Animal Care and Use Committee and approved by the Ethic Committee of the Health Sciences University of Hokkaido (No.165). Twenty retired Wistar rats (10–12 months old female, weight range 280–320 g) were used to evaluate in vivo effect of bone formation by PDDM.

#### 2.5.1. Surgical Procedure

**Figure 2:**
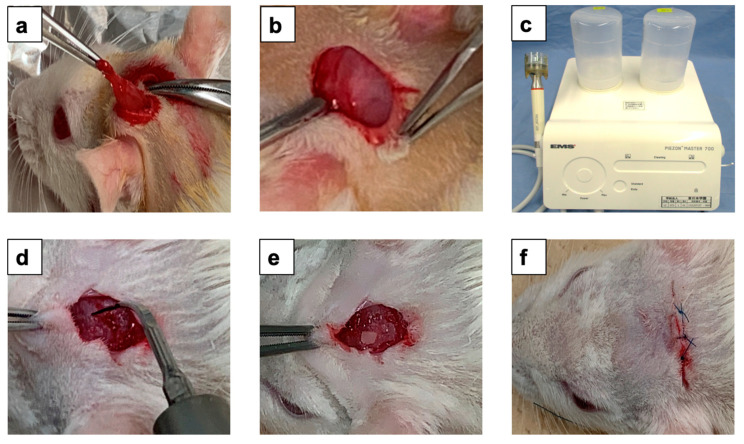
Schematic representation of ultrasonic scaler scratching design and PDDM graft in experimental group. (**a**) Periosteum removed from parietal bone after the elevation of scalp, (**b**) Exposed parietal bone, (**c**) Piezoelectric ultrasonic scaler unit, operated at 24–32 kHz, 8–12 Watt for 1 min, (**d**) Exposed parietal bone treated by ultrasonic scaler tip for 1 min, (**e**) PDDM onlay graft on scratched skull and (**f**) Skin sutured with 4/0 polyglactin resorbable sutures. PDDM: Partially demineralized dentin matrix.

Schematic representation of the surgical experimental design shown in Figure 2. First, the rats were divided randomly into two groups, as follows: control group, onlay PDDM block on the exposed parietal bone without periosteum (n = 10) and experimental group, onlay graft of PDDM with ultrasonic scaler tip scratch on rat skull (n = 10). All rats were fed with a standard diet during the experimental period. Wistar rats were subjected to intraperitoneal anesthesia with pentobarbital sodium (4 mg/100 g body weight). Surgical operations were performed under sterile conditions. Following general anesthesia, prior to surgery, the skull skin was shaved. A skin incision on the skull was made over the linea media. An incision allowing reflection of a full-thickness flap in the anterior-posterior direction was made in the scalp in the sagittal plane. An elevator was used to lift the skull skin and the periosteum was removed. In the control group, animals received PDDB on the skull parietal bone without periosteum. In the experimental group, animals received ultrasonic scaler tip scratch for 1 min before PDDM graft. The ultrasonic scaler unit (Piezon Master 700, Shofu Inc., Kyoto, Japan) was operated at a frequency of 24–32 kHz and an output of 8–12 W, with an effective mechanical force of around 5 Newton (5 N) for 1 min using an interdental scaler tip. During this process, extreme care was taken not to damage the graft site. All rats in each group were treated as indicated above. All surgical procedures were performed by the same surgeon (SD). The skull skin was sutured with 4/0 polyglactin resorbable sutures. Chloromycetin ointment were used for surgical incision in all animals after the surgery.

At 4 and 8 weeks after the onlay grafts, the rats were sacrificed (5 rats as the control group and 5 rats as the experiment group at each time point) by cervical dislocation under an anesthetic overdose (ketamine at a dose 2–3-fold higher than the anesthetic dosage). After the incision of head skin, a surgical drill attached to an electrical hand motor piece was used to harvest the skull bone with PDDM and skin. The specimens were prepared for histological examination (Figure 2).

#### 2.5.2. Histological Examination

##### Hematoxylin and Eosin (HE) Staining

At time intervals, the bone specimens were fixed in 10% neutral phosphate-buffered formalin solution for 1 week and decalcified with 10% formic acid for 4 weeks and rinsed overnight with running water. All the samples were dehydrated using ethanol (50–100%) and processed for paraffin embedding (Vacuum Rotary, VRX-23, Mitsubishi, Tokyo, Japan). Later, a 5 µm thickness of histological sections were prepared by a microtome (Yamato Rom 380, Tokyo, Japan) and stained with hematoxylin and eosin (HE) (Wako, Osaka, Japan). The HE-stained sections were examined using an optical microscopy (Nikon Eclipse 80i, Nikon, Tokyo, Japan) for histological evaluation.

##### Tartrate-Resistant Acid Phosphatase (TRAP) Staining

Slides were put into xylene for 5 min three times, shook in 100%, 90%, 80% and 70% ethanol for 2 s two times and washed in the water for 10 min. Next, the reaction solution was made by 5 mg of Naphthol AS-BI (N2250-1G, Sigma-Aldrich, St. Louis, MO, USA), 0.15 mL of N.N-Dimethyl Formamide (Guaranteed Reagent 10344 Kanto Chemical Co. Inc., Tokyo, Japan), 25 mL of D.W., 0.2 mol acetate buffer (pH = 5.4) and 0.383 g of L (+)-Tartaric Acid (M6T3632 Nacalai Tesque, Inc., Kyoto, Japan). After that, the solution was kept on rotation for 10 min, and the pH was adjusted to 5.2 by 1 mol/L-Sodium Hydroxide Solution (L8E4959 Nacalai Tesque, Inc., Kyoto, Japan). After filtering the solution with filter paper, the slides were kept in the solution and warm in hot water bath for 30 min. The TRAP-stained sections were examined using an optical microscopy for histological evaluation.

### 2.6. Statistical Analysis

All numerical data are presented as the mean ± standard deviation (SD). The statistical significance of the change in response was assessed by a paired t-test. Differences were considered significant at p < 0.05. The statistical analysis was performed using a Windows computer with the SPSS software version 19 (IBM, Armonk, New York, NY, USA).

## 3. Results

### 3.1. Morphological Characterization by Field Emission Scanning Electron Microscope (FE-SEM)

**Figure 3:**
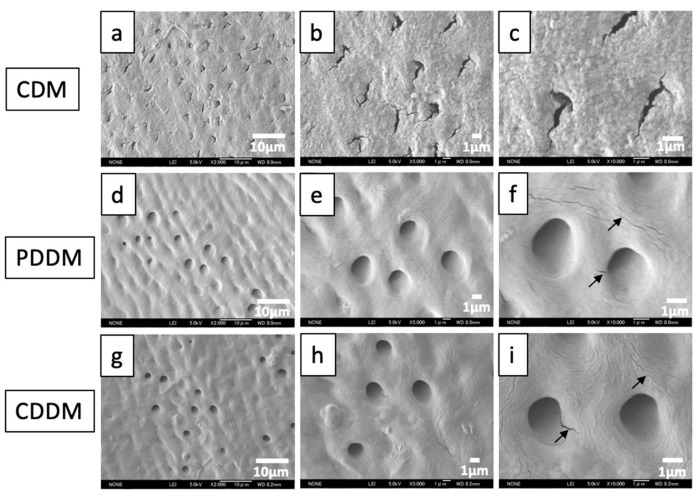
FE-SEM photographs of CDM (**a**–**c**), PDDM (**d**–**f**) and CDDM (**g**–**i**). (**a**–**c**) CDM: Mineralized dentin with irregular surface. Almost closed dentinal tubes affected by machine cutting. (**d**–**f**) PDDM: Opening of dentinal tubes and smooth surface. Micro-cracks (arrows). (**g**–**i**) CDDM: Opening of dentinal tubes and smooth surface. Micro-cracks (arrows). CDM: Calcified dentin matrix, PDDM: Partially demineralized dentin matrix, CDDM: Completely demineralized dentin matrix.

The topographical analysis of CDM, PDDM and CDDM was shown in Figure 3. The SEM images of CDM showed almost closed dentinal tubes affected by the machine cutting and mineralized surface (Figure 3a–c). PDDM and CDDM exhibited a homogenous dentin surface structure with well exposed tube-type pores (Figure 3d–i). The dentinal tubes of PDDM and CDDM were wider with dentinal hole diameters at approximately 1–2 μm on the dense collagen matrix surface (Figure 3e,f,h,i). Several nano-microcracks and damages due to ultrasonically 2% HNO_3_-demineralization were also found in the surfaces of both PDDM (Figure 3f) and CDDM (Figure 3i). 

### 3.2. Dissolved Efficiency of Dentin Blocks

**Figure 4:**
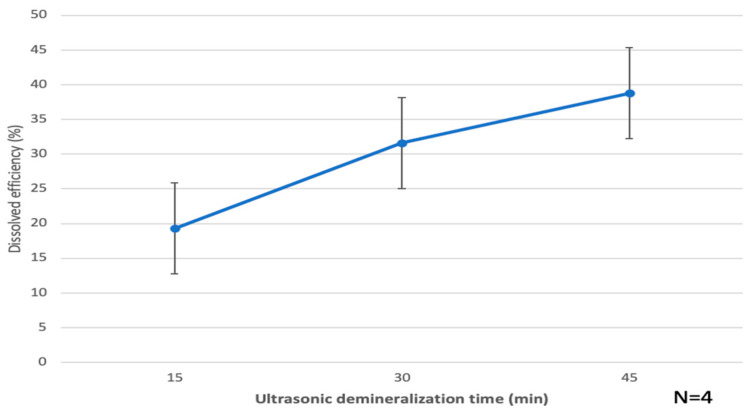
Dissolved efficiency of CDM blocks by ultrasonic demineralization in 1.0 L of 2% HNO_3_ solution. Demineralized dentin for 30 min: named as PDDM. CDM: Calcified dentin matrix, PDDM: Partially demineralized dentin matrix.

The dissolved efficiency of CDM blocks at 15 min, 30 min and 45 min were 19.4% ± 5.7, 32.3% ± 5 and 39.1% ± 6.7, respectively (Figure 4). Dentin block demineralized ultrasonically for 30 min, so named PDDM block, showed 32.3%. The dissolved efficiency at 45 min was about twice of that at 15 min (Figure 4).

### 3.3. Absorption Characteristics and Microstructure of Dentin by Collagenase Digestion

**Figure 5:**
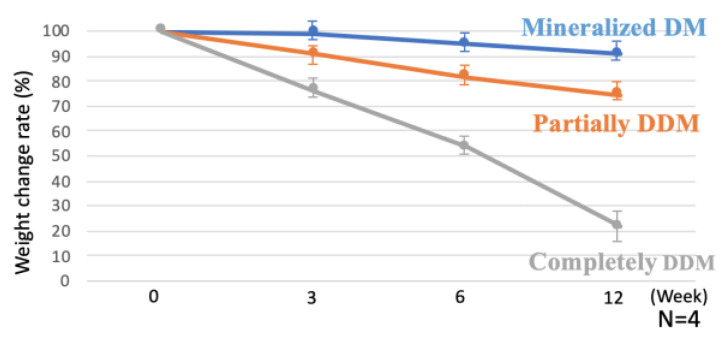
Dentin absorption by collagenase digestion. Weight change ratio of PDDM showing 91.2%, 82.4% and 74.5% at 3, 6 and 12 weeks after collagenase digestion. Weight-decreasing rate of CDM, PDDM and CDDM blocks at 12 weeks were 9.2%, 25.5% and 78.3%, respectively. There was a significant difference in weight change rate between CDM, PDDM and CDDM at 3, 6 and 12 weeks. CDM: Calcified dentin matrix, PDDM: Partially demineralized dentin matrix, CDDM: Completely demineralized dentin matrix.

Figure 5 shows dentin absorption characteristics by collagenase digestion. Weight change rates for CDM and PDDM gradually decreased with increasing the digestion time, while those for CDDM significantly decreased. Especially, at 12 weeks, weight change rates were 9.2% ± 5.5 for CDM, 25.5% ± 4.9 for PDDM and 78.3% ± 8.4 for CDDM, respectively. 

**Figure 6:**
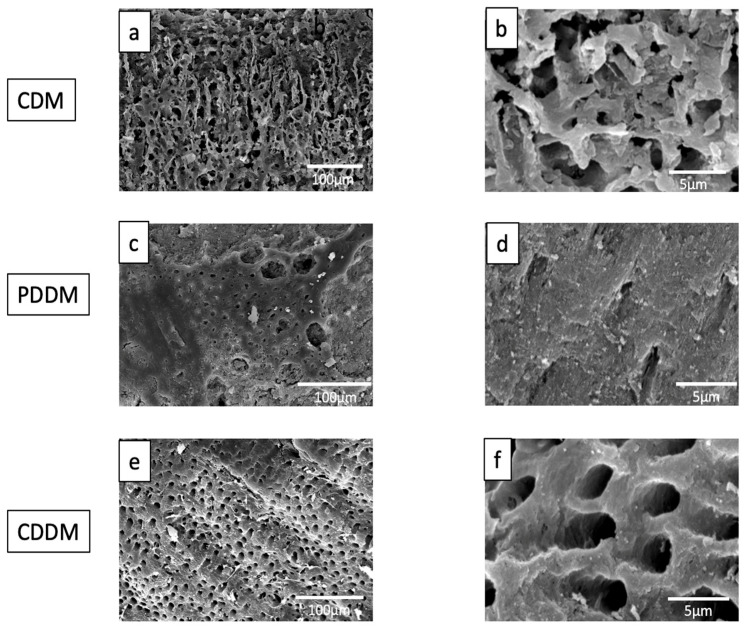
SEM photographs of CDM, CDDM and PDDM after collagenase digestion for 12 weeks. (**a**,**b**) CDM: Trabecular and irregular structure. (**c**) PDDM: Various crater-like round spaces. (**d**) PDDM: dentinal tube-spaces with scale-like surface. (**e**,**f**) CDDM: Uneven surface with wide dentinal tube spaces and irregular bulk matrix. CDM: Calcified dentin matrix, PDDM: Partially demineralized dentin matrix, CDDM: Completely demineralized dentin matrix.

At 12 weeks after collagenase digestion, microstructures for three kinds of dentin block were shown in Figure 6. Increased collagen network degradation was recognized after the collagenase digestion. PDDM and CDDM exhibited irregular dentin surface structure with exposed dentinal tubules of different sizes. Loose inter- and peritubular dentin fiber bundles were observed (Figure 6c–f).

### 3.4. Histological Findings in PDDM Onlay Graft on Non-Scratched or Scratched Skull without Periosteum

**Figure 7:**
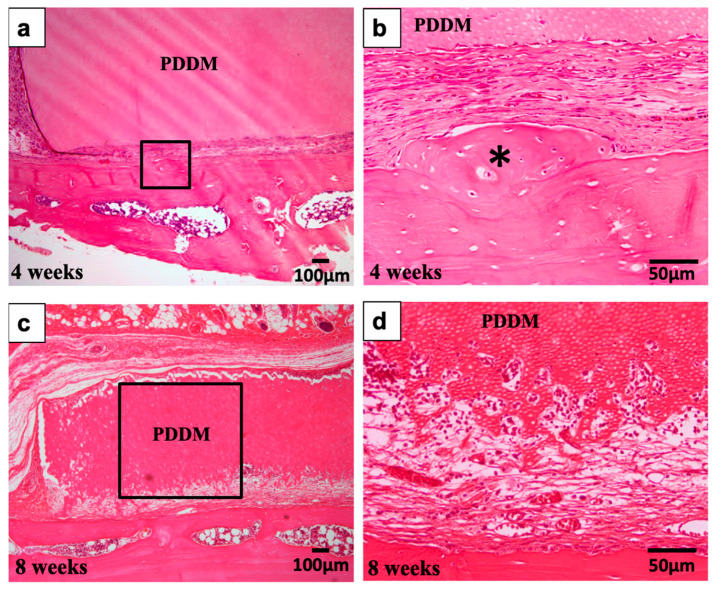
Histological images of non-scratched skull without periosteum at 4 weeks (**a**,**b**) and 8 weeks (**c**,**d**) as controls. (**a**) Fibrous connective tissues between PDDM block and Skull. (**b**) Higher magnification of frame in (**a**). Locally, reactive bone formation (*). Acellular original bone indicating necrotic bone. (**c**) No bony bridge. Note: marrow space-closing. Absorption of bottom surface of block. (**d**) Higher magnification of frame in (**c**). Wavy surface of PDDM showing a different structure from skull surface. PDDM: Partially demineralized dentin matrix.

In the PDDM on non-scratched parietal bone without periosteum as the control group, bony bridge formation was not found between PDDM and skull at both 4 and 8 weeks after the grafts (Figure 7). PDDM was encapsulated by fibrous connective tissues, while reactive bone formation was seen locally on skull at 4 weeks (Figure 7a,b). New bone was divided from acellular original bone, indicating necrotic bone (Figure 7b). Bone marrow space-opening was not found at 4 and 8 weeks (Figure 7a,c). In the bottom of PDDM, active absorption of PDDM was observed at 8 weeks, while multinucleated giant cells did not appear clearly (Figure 7c,d).

**Figure 8:**
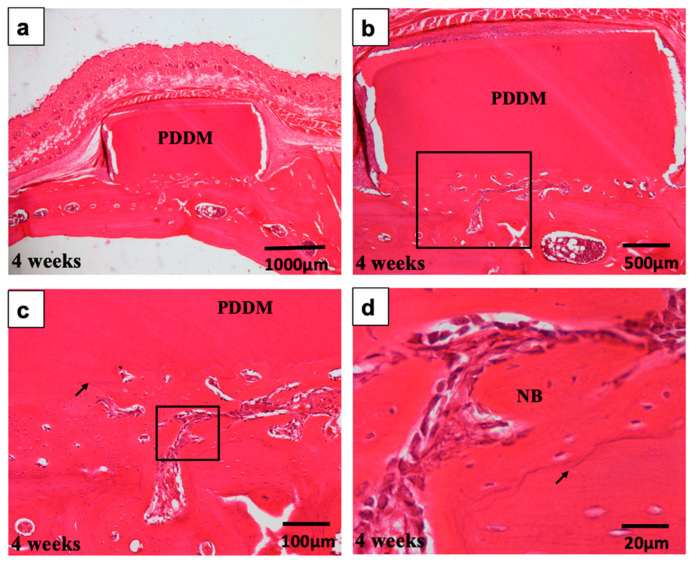
Histological images of scratched skull without periosteum at 4 weeks. (**a**) Acellular PDDM block connected with skull. (**b**) New bone formation widely between PDDM and skull. (**c**) Higher magnification of frame in (**b**). PDDM divided from new bone by the presence of a linear cementum line (arrow) between them. (**d**) Higher magnification of frame in (**c**). New bone with osteoblast-lining divided from skull by cementum line (arrow). PDDM: Partially demineralized dentin matrix.

In the experimental group, the PDDM on scratched parietal bone without periosteum was connected directly with newly formed bone, and some parts were encapsulated by lose mesenchymal tissues with spindle-type cells (Figure 8a,b). At 4 weeks, bony bridge was observed between PDDM and scratched skull bone (Figure 8). New bone could be easily divided from the grafted PDDM by the presence of a linear cementum line between them (Figure 8c). Bone formation occurred mainly at the recipient–graft interface near marrow space opening. Woven bone formation occurred locally with osteoblasts overlying it, while osteoblast differentiation did not occur on the upon area of PDDM (Figure 8c,d).

**Figure 9:**
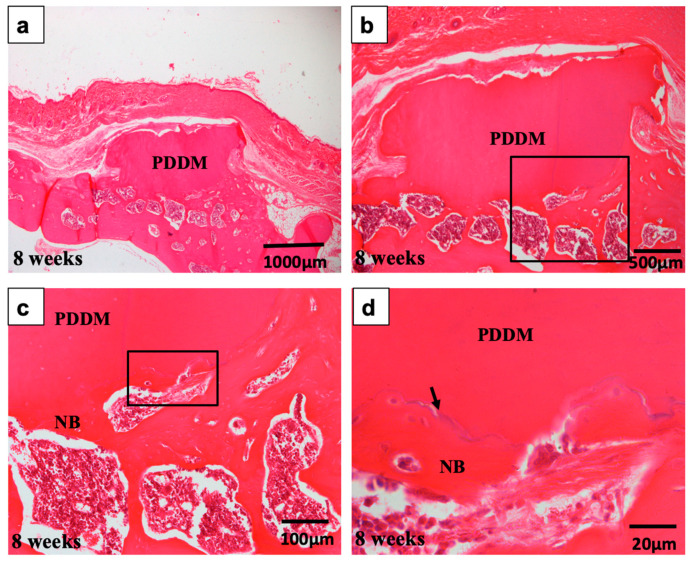
Histological images of scratched skull without periosteum at 8 weeks. (**a**) Acellular PDDM block connected with skull. Absorption of upper and lateral sides of block. (**b**) New bone formation widely between PDDM and skull. (**c**) Higher magnification of frame in (**b**). New bone with red marrow. (**d**) Higher magnification of frame in (**c**). PDDM divided from new bone by the presence of a linear cementum line (arrow). PDDM: Partially demineralized dentin matrix.

**Figure 10:**
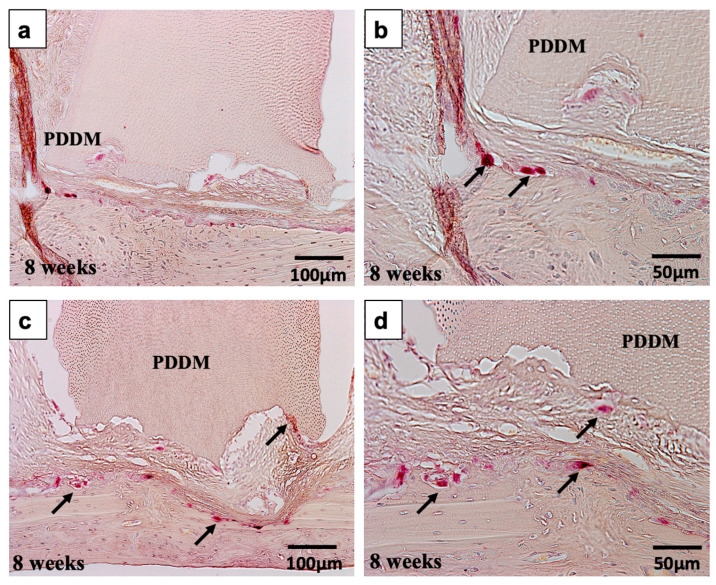
TRAP-staining images of scratched skull without periosteum at 8 weeks. Sections (**a**,**b**) and (**c**,**d**) were derived from different blocks. TRAP-positive cells (arrow) appeared predominantly nearby skull, compared with sides of PDDM block. PDDM: Partially demineralized dentin matrix.

At 8 weeks, gross morphological changes of the PDDM were observed, with the presence of multiple deep trenches on the dense outer plate (Figure 9). The PDDM was collapsed and the edge of it became much rounder, although the majority of PDDM remained non-absorbed (Figure 9a,b). Empty lacunae spaces were found in the vicinity of the outer cortical plate (Figure 9c). In the case of bony bridge formation, marrow space-opening from the outer cortical plate was clearly found at 4 and 8 weeks. In addition, TRAP staining images revealed the presence of osteoclasts nearby the skull bone not PDDM at 8 weeks after implantation (Figure 10).

### 3.5. TRAP-Staining Images of Scratched Skull without Periosteum 

Figure 10 showed TRAP-staining images of scratched skull without periosteum at 8 weeks. Sections (Figure 10a,b) and (Figure 10c,d) were derived from different blocks. TRAP-positive cells appeared predominantly nearby skull, compared with sides of PDDM block.

## 4. Discussion

Dental pioneers have begun to recycle patient-own dentin materials for clinical use in the 21st century. Generally, adult-senior stage patients lose teeth and alveolar bone with the periosteum due to periodontal diseases and require vertical and/or horizontal augmentation for implant rehabilitation. Until now, almost all in vivo studies were conducted in bone defect (inlay) models by using DDM particles, not onlay. Additionally, the mechano-chemical properties of human dentin blocks have not been sufficiently investigated. In this study, therefore, the chemical properties of human dentin blocks were characterized, and human PDDM blocks were grafted onto the skull without a periosteum of 10–12-month-old rats (adult-senior stage) as a challenging onlay model. 

Extensive studies have been conducted to evaluate the geometry, biochemical nature, biocompatibility and osteoinductivity of DDM scaffolds [7,8,10,19,24]. It is well-known that porous graft materials promote bone formation better than the dense type [25]. A non-porous solid material acts as a biomaterial wall, as it inhibits both cellular and capillary invasion [26]. Human dentin has a dense structure, like cortical bone. Although dentin or bone block provides the necessary support and stability, it does not allow for sufficient diffusion of growth factors. Previous studies have confirmed that bone regeneration using block or non-porous biomaterial would require considerably more time for complete anatomical and functional recovery [27]. Dentin blocks modifications by including porosities and partial demineralization influence the cellular response and bone tissue regeneration rate [19]. Semi-rigid dentin scaffolds prepared using partial demineralization, i.e., PDDM, provide better osteogenic activity compared with calcified and completely demineralized dentin [28]. In addition, ultrasonic scaler treatment could bring about surface modification and morphological changes to the hard tissues, such as bone and dentin, in a time-dependent manner [29]. This mechano-chemical modification of the dentin block should influence the new bone regeneration rate. Therefore, we considered the simultaneous mechano-chemical processing of the dense block-type human dentin modified by ultrasonic 2% HNO_3_-demineralization for 30 min and the cortical bone (skull) scratched by an ultrasonic tip. In this study, the FE-SEM results provided evidence that ultrasonic irradiation enhanced the physical nature of the physiological cracks and the creation of new nano-micro cracks and damage, along with attenuating the already present physiological microcracks with destroyed fibers. The changes in the surface morphology correlated strongly with the migration and proliferation of the mesenchymal cells necessary for bone tissue formation, as previously demonstrated [30]. 

The demineralization process of dentin block increases the release and bioavailability of matrix-associated proteins, especially BMPs, thus rendering these grafts osteoinductive [31]. It is well known that PDDM particles have superior bone regenerative capacity compared to CDM particles [7]. Preparation of human PDDM by partial demineralization has been found to be effective in ectopic models [10,32] and in bone defect models [19,23]. A 2% HNO_3_-demineralization process for human PDDM particles creates surface decalcifications with better bone regeneration due to greater cellular attachment, including osteoblasts and osteoprogenitor cells on the PDDM surface [23]. Human root-form dentin block was demineralized ultrasonically in 2% (0.31 N) HNO_3_ for 30 min, and the PDDM block regenerated new bone actively in the critical-sized bone defect of the iliac crest of adult sheep at 2 and 4 months [19]. Clinically, the time and speed of bone surgery are very important factors especially for immediate graft, and thus 30 min-partial demineralization in 2% HNO_3_ was used in the present study.

Ultrasonic demineralization should create more micro- and nano-cracks to increase the effective porous volume for better cellular attachment, although stirring treatment has conventionally been used for demineralization. The interconnected porous volume implies an increase in the body fluid contact surface area and growth factor release [29]. Moreover, partial demineralization of human dentin blocks could maintain mechanical properties and preserve calcium-binding proteins such as osteocalcin after the demineralization process. Through histological visualization, we observed active bone ingrowth in between the PDDM and modified scratched skull bone at 4 weeks after grafting (Figure 8). The simultaneous interconnected structure modifications with higher total available surface area might be the right design concept for improving dentin materials. Regarding the development and application of supersonics, ultrasonic echo, ultrasonic knife, ultrasonic microscope, ultrasonic cleaning, ultrasonic sterilization and ultrasonic therapy for fracture are widespread in the medical and dental fields. Supersonic waves with a frequency greater than 20 kHz can induce bubble cavitation and make hot spots. In these hot spots, many chemical reactions will be activated by the formation of radical groups and the locally rising temperature. The surface structure design of biomaterials originating from human teeth following supersonic treatment can easily produce new biomimetic biomaterials which control the bio-absorption rate and the adsorption ability for proteins or cells. In our previous reports, the dissolution efficiency of hydroxyapatite (HAp) products by supersonic treatment drastically increased with time, depending on the porosity and the concentration of HNO_3_ aqueous solution. The dissolution efficiencies by the supersonic treatment for a very short time were much higher than those provided by stirring treatment [32]. For even dense HAp products, enhancement of micro-pores and propagation of micro-cracks were recognized with supersonic technique at 120 W, 38 kHz and pH 1.0 for 10–20 min [32]. In another study, using DDM granules obtained by supersonic dissolution at 120 W and 38 kHz, as supersonic time increased, the asperity on the surfaces of granules became outstanding, due to the elution of mineral components [29,33]. The specific surface areas increased a little more during demineralization and the physical structure of residual dentinal tubules, such as tube diameter and length, were clearly observed. Body fluid will smoothly permeate through the micro-cracks if these DDM granules are implanted into a living body. The partial supersonic dissolution treatment may be used as a convenient and effective preparation technology to produce bio-absorbable and bioactive materials. 

Both the matrix digestion by host tissue-derived enzymes and the cellular phagocytosis by multinucleated giant cells are dynamic processes involved in the bio-absorption of dentin blocks [34,35]. Previous studies have confirmed the bio-absorption of the partially demineralized root dentin matrix by cellular phagocytosis in vivo [28]. The supersonic demineralization of the dentin scaffold leads to exposure of the superficial demineralized dentin collagen matrix. The exposed matrices are bio-absorbed by enzymes under different physiological and pathological conditions [35]. Although collagenolytic activity identified from bacteria may contribute to the degradation of dentinal matrices [36], recent studies suggest that different types of enzymes play an equally important role in dentin bio-absorption [37,38,39]. In this study, in vitro absorption of CDM, PDDM (30 min-demineralization) and CDDM (3 day-demineralization) by collagenase digestion was successfully evaluated, as shown in Figure 5. The exposed collagen matrices of PDDM and CDDM were degraded and decreased more than CDM by collagenase, while CDM blocked collagenase digestion (Figure 6). Meanwhile, the results of the in vitro collagenase digestion test suggested that the degradation rate of PDDM, compared to CDM, could sustain tissue-repairing space, which plays an important role in the early osteogenic process [28]. To the best of our knowledge, this study described for the first time the enzymatic behavior of human PDDM block in vitro.

In the PDDM on scratched parietal bone without a periosteum, a bony bridge between PDDM block and dense skull was formed widely or locally. More importantly, marrow-space openings from the outer cortical plate were found at 4 and 8 weeks (Figure 7 and Figure 9). From the histological points of view, it was considered that the supply of marrow cells from original bone had a highly important role for bony bridge formation and augmentation on the highly calcified skull. Altogether, these results demonstrate that ultrasonic scaler tip treatment (scratching) into the compact parietal bone of 10–12-month-old rats might contribute to the marrow-space opening, and, thus, undifferentiated mesenchymal cells and osteogenic cells in bone marrow could differentiate into osteoblasts near the bottom of the PDDM in this vertical augmentation model. Finally, human PDDM block could achieve bony bridge formation under the scratched skull condition with bone marrow-space opening in this vertical augmentation model without a periosteum.

As for the limitation in this study, bone induction did not occur on the upper sites of PDDM block until 8 weeks. We believe the reasons were due to the small amount of BMP-2 (6.2 ng/mg DDM) in human DDM [6], the release of growth factors from DDM dense block, and non-osteogenic tissue conditions without periosteum in this model. In the near outlook, PDDM with BMP-2 and structurally modified PDDM will be grafted for totally successful bone augmentation.

## 5. Conclusions

Human tooth-derived dentin blocks were chemically characterized and the changes of the original bone and PDDM block were evaluated on 10–12-month-old rat skulls without periosteum as vertical augmentation models. The SEM images of dentin blocks after the collagenase digestion revealed degradation of collagen network in vitro. The bone marrow-space opening from original bone was found in the bony bridge formation between the human PDDM block and dense rat skull of adult-senior stage at 4 and 8 weeks. On the other hand, in the cases of the marrow-space closing, the bony bridge was not formed. The results indicated that the ultrasonic scratching into the compact parietal bone might greatly contribute to the supply of bone marrow cells and the formation of bony bridge in the vertical augmentation model without a periosteum.

## Data Availability

Data sharing is not applicable to this article.
